# *In silico* characterization of *bla*_NDM_-harboring plasmids in *Klebsiella pneumoniae*

**DOI:** 10.3389/fmicb.2022.1008905

**Published:** 2022-11-23

**Authors:** Zhu Zeng, Lei Lei, Linman Li, Shengni Hua, Wenting Li, Limei Zhang, Qiuping Lin, Zhixiong Zheng, Jing Yang, Xiaohui Dou, Luan Li, Xiaobin Li

**Affiliations:** ^1^Department of Respiratory Diseases, The First Affiliated Hospital of College of Medicine, Zhejiang University, Hangzhou, China; ^2^Department of Cadre Health Care, Guizhou Provincial People's Hospital, Guiyang, China; ^3^Health Management Center, The First Affiliated Hospital of Chongqing Medical University, Chongqing, China; ^4^Department of Radiation Oncology, Zhuhai People’s Hospital (Zhuhai Hospital Affiliated With Jinan University), Zhuhai, China; ^5^Zhuhai Precision Medical Center, Zhuhai People’s Hospital (Zhuhai Hospital Affiliated With Jinan University), Zhuhai, China; ^6^Department of Pulmonary and Critical Care Medicine, Zhuhai People's Hospital (Zhuhai Hospital Affiliated With Jinan University), Zhuhai, China; ^7^Health Management Center, Zhuhai People’s Hospital (Zhuhai Hospital affiliated With Jinan University), Zhuhai, China; ^8^Department of Oncology, Jiangsu Cancer Hospital & Jiangsu Institute of Cancer Research & The Affiliated Cancer Hospital of Nanjing Medical University, Nanjing, China

**Keywords:** *Klebsiella pneumoniae*, plasmid, *bla*_NDM_, replicon types, conjugative transfer region

## Abstract

*Klebsiella pneumoniae* is a primary culprit of antibiotic-resistant nosocomial infections worldwide, and infections caused by NDM-producing strains are a major threat due to limited therapeutic options. The majority of *bla*_NDM_ cases occur on plasmids; therefore, we explored the relationships between plasmids and *bla*_NDM_ genes in *K. pneumoniae* by analyzing the variants of *bla*_NDM_, replicon types, conjugative transfer regions of 171 *bla*_NDM_-harboring plasmids from 4,451 *K. pneumoniae* plasmids. Of the nine identified *bla*_NDM_ variants, *bla*_NDM-1_ (73.68%) and *bla*_NDM-5_ (16.37%) were the most dominant. Over half of the *bla*_NDM_-harboring plasmids of *K. pneumoniae* were classified into IncF plasmids. IncX3 single-replicon plasmids (46–57 kb) carried genes encoding relaxases of the MOB_P_ family, T4CP genes of the VirD4/TraG subfamily, and VirB-like T4SS gene clusters, which were mainly geographically distributed in China. We found 10 *bla*_NDM_-harboring IncN plasmids (38.38–63.05 kb) carrying the NW-type origin of transfer (*oriT*) regions, genes coding for relaxases of MOB_F_ family, genes encoding T4CPs of the TrwB/TraD subfamily, and Trw-like T4SS gene clusters, which were also mainly geographically distributed in China. Moreover, we identified 21 IncC plasmids carrying *bla*_NDM-1_ (140.1–329.2 kb), containing the A/C-type *oriT*s, genes encoding relaxases of MOB_H_ family, genes encoding T4CPs belonging to TrwB/TraD subfamily, and Tra_F-like T4SS gene clusters. The *bla*_NDM_-harboring IncC plasmids were widely geographically distributed all over the world, mainly in the United States, China and Viet Nam. These findings enhance our understanding of the diversity of *bla*_NDM_-harboring plasmids in *K. pneumoniae*.

## Introduction

*Klebsiella pneumoniae* is a significant cause of nosocomial infections such as pneumonia, bloodstream infections, urinary tract infections, and septicemias (Pitout et al., 2015; Bengoechea and Sa Pessoa, 2019). *Klebsiella pneumoniae* represents one of the most concerning pathogens known for its high frequency and diversity of antimicrobial resistance (AMR) genes (Navon-Venezia et al., 2017; Wyres and Holt, 2018), and it has been classified as an ESKAPE organism (De Oliveira et al., 2020). The emergence and spread of carbapenem-resistant *K. pneumoniae* have become severe medical problems worldwide (Navon-Venezia et al., 2017). Resistance to carbapenems in *K. pneumoniae* involves diverse mechanisms, e.g., production of carbapenemases (e.g., KPC, NDM, and OXA-48-like), alterations in outer membrane permeability and the upregulation of efflux systems (Pitout et al., 2015).

New Delhi metallo-β-lactamase (NDM), belonging to Ambler class B β-lactamase, has the ability to hydrolyze all β-lactam antibiotics (including carbapenems) except the monobactam aztreonam (Nordmann et al., 2011). NDM-1 was first reported in a *K. pneumoniae* isolate recovered in a Swedish patient who traveled to New Delhi in 2008 (Yong et al., 2009). According to the records of the Beta-lactamase database (BLDB; Naas et al., 2017) on September 8th, 2022, more than 40 variants of NDM have been identified so far. A variety of infections caused by NDM-producing Enterobacterales strains are associated with inferior prognosis and high mortality, especially in high-risk immunocompromised patients (Guducuoglu et al., 2018). NDM-producing Enterobacterales clinical isolates, mainly *K. pneumoniae* and *Escherichia coli*, have been found worldwide, with a higher prevalence in the Indian subcontinent, the Balkans, and the Middle East (Albiger et al., 2015; Wu and Feng, 2019).

Antimicrobial resistance (AMR) in carbapenem-resistant Enterobacterales (CRE) strains is often encoded by the plasmid-borne genes (Rozwandowicz et al., 2018). Plasmids, especially conjugative plasmids, play an essential role in mediating horizontal gene transfer (HGT) and dissemination of AMR (Jiang et al., 2020). The conjugative transfer regions of conjugative plasmids typically comprise four key modules, including origin of transfer (*oriT*) region, gene encoding relaxase, gene encoding type IV coupling protein (T4CP), and gene cluster for the bacterial type IV secretion system (T4SS) apparatus (de la Cruz et al., 2010). The relaxase initiates the bacterial conjugation by recognizing and cleaving the *oriT* of the plasmid in a site-specific manner, forming a relaxosome (Llosa et al., 2002; Carballeira et al., 2014). Currently, nine types of plasmid-borne *oriT*^1^ and eight main relaxase families^2^ have been identified (Li et al., 2018). Conjugation requires a pilus, which is assembled by T4SS, to connect the donor and the recipient strains (de la Cruz et al., 2010). Currently, five main types of T4SS gene clusters are defined, including 18 different kinds of systems^3^ (Bi et al., 2013). The T4CP connects the relaxosome to T4SS, which is required for conjugation, and currently, two main subfamilies of T4CPs^4^ exist (Li et al., 2018).

Studies on the comprehensive analysis of *bla*_NDM_-harboring plasmids and their conjugative transfer regions in *K. pneumoniae* are scarce. In this work, we executed *in silico* typing and comparative analysis of *bla*_NDM_-harboring plasmids of *K. pneumoniae* using the bacterial plasmids available in the NCBI GenBank database. We systematically analyzed the variants of *bla*_NDM_, replicon types, phylogenetic patterns, and conjugative transfer regions of the *bla*_NDM_-positive plasmids of *K. pneumoniae*. This study provides deep insights into the characteristics and diversity of *bla*_NDM_-harboring plasmids in *K. pneumoniae* and further emphasizes their role in dissemination of resistance genes.

## Materials and methods

### Plasmid sequences from the NCBI database

The GenBank Genome database (Benson et al., 2018) collect all the plasmids belonging to *K. pneumoniae*.^5^ A total of 4,451 plasmids (without duplicates) of *K. pneumoniae* (Supplementary Table S1) were downloaded on April 26th, 2022. Files in FASTA DNA format of the 4,451 plasmids were downloaded in batches into our Linux-based server.

### Identification of the *bla*_NDM_-harboring plasmids of *Klebsiella pneumoniae*

The β-lactamase genes of the plasmids of *K. pneumoniae* were identified applying the ResFinder software, standalone version 4.1 (Bortolaia et al., 2020), with the minimum coverage of 60%, minimum identity of 90%, and species of “Klebsiella.” The term “*bla*_NDM_” was used to search in the “Resistance gene” list of the ResFinder results in order to judge the *bla*_NDM_-harboring plasmids of *K. pneumoniae* and identify the variants of the *bla*_NDM_ genes. For some *bla*_NDM_-harboring plasmids, the variants of *bla*_NDM_ were not determined by the ResFinder software; instead, they were submitted to the CARD database^6^ (Alcock et al., 2020) and the Beta-lactamase database (BLDB; Naas et al., 2017) for further analysis.

### Replicon typing of the *bla*_NDM_-harboring plasmids of *Klebsiella pneumoniae*

Replicon typing of the *bla*_NDM_-harboring plasmids was executed *via* the PlasmidFinder software (Carattoli and Hasman, 2020). Then, selecting the database “Enterobacteriales,” the FASTA-formatted DNA files were analyzed and classified in batches by using the PlasmidFinder tool version 2.0.1, with a minimum coverage cut-off of 60% and minimum identity cut-off of 95%. The database version was updated on November 29th, 2021.

### Phylogenetic cladogram of the *bla*_NDM_-harboring plasmids of *Klebsiella pneumoniae*

The files of the *bla*_NDM_-harboring plasmids of *K. pneumoniae*, in GenBank format, were downloaded in batches using two Bioperl modules (Bio::SeqIO and Bio::DB::GenBank). Plasmid files containing protein sequences were compiled from the plasmid files in GenBank format through the Bioperl/Bio::SeqIO module. Phylogenetic cladogram based on the presence/absence of orthologous gene families of all the *bla*_NDM_-harboring plasmids of *K. pneumoniae* were constructed. First, a binary gene presence/absence matrix was built using OrthoFinder software (Emms and Kelly, 2019), and subsequently a hierarchical cluster result was generated by PAST3 (Hammer et al., 2001) and eventually displayed by iTOL (Letunic and Bork, 2016).

### Geographic location and host ST types of the *bla*_NDM_-harboring plasmids in *Klebsiella pneumoniae* strains

Information about geographic location of *bla*_NDM_-harboring plasmids and its host strains were extracted from the files of the *bla*_NDM_-harboring plasmids in GenBank format. Table containing the correspondence between strains and plasmids of *K. pneumoniae* were downloaded from the GenBank.^7^ The *bla*_NDM_-harboring plasmid-matched host *K. pneumoniae* strains were collected, and their DNA FASTA sequences were downloaded in batch using the Bioperl. The MLST software (Larsen et al., 2012) version 2.0.9 was downloaded from the website^8^ and installed on the Linux platform. The genomes of *K. pneumoniae* strains were analyzed in batch using MLST software.

### Characterization of the conjugative transfer regions of *bla*_NDM_-harboring plasmids

Files in GenBank format of the *bla*_NDM_-harboring plasmids in *K. pneumoniae* were analyzed in batches using oriTfinder software (local version; Li et al., 2018) to identify the presence/absence of *oriT*s, relaxase-coding genes, T4CP-coding genes, and gene clusters for T4SS. Furthermore, the types of *oriT*s, relaxases, T4CPs, and T4SSs toward the plasmids were determined based on the exhibition of the oriTDB database^9^ (Li et al., 2018). In addition, the types of gene clusters for T4SS were classified based on the SecReT4 database^10^ (Bi et al., 2013).

### Bipartite network construction, clustering and visualization of the *bla*_NDM_-harboring plasmids of *Klebsiella pneumoniae*

The bipartite network was constructed based on all the *bla*_NDM_-harboring plasmids of *K. pneumoniae* using the AccNet software using default parameters (Lanza et al., 2017). The obtained network files including nodes, edges and clusters were then imported into the Cytoscape software (Shannon et al., 2003) for visualization. We displayed the relative genomic content of each plasmid by making the diameter of each node proportional to its degree.

## Results

### Variants of *bla*_NDM_ genes in the *bla*_NDM_-harboring plasmids of *Klebsiella pneumoniae*

**Figure 1:**
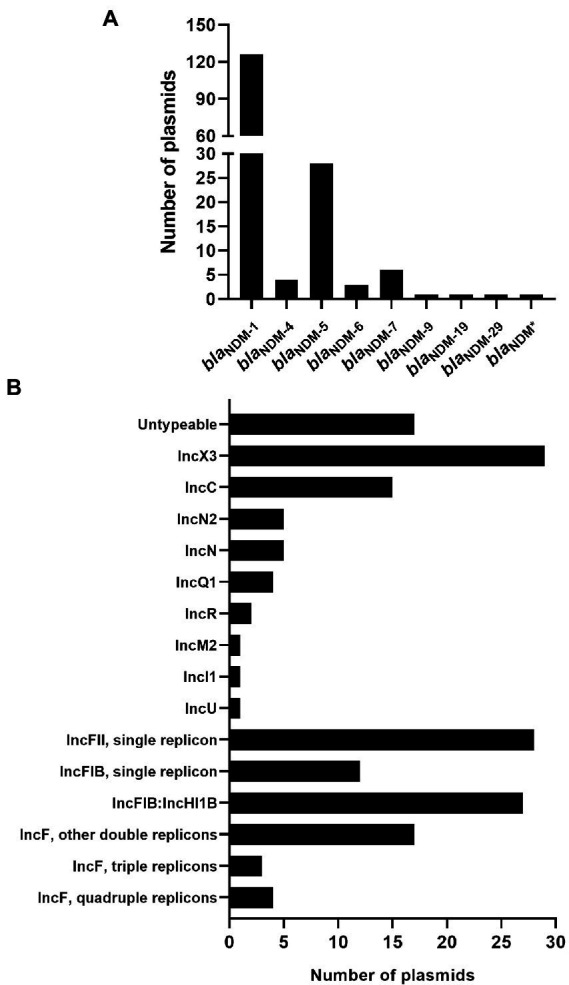
Characteristics of the 171 *bla*_NDM_-harboring plasmids of *Klebsiella pneumoniae*. **(A)** Histogram of number of variants of *bla*_NDM_ genes among the 171 *bla*_NDM_-harboring plasmids of *K. pneumoniae*. *bla*_NDM*_ representing an unknown variant of *bla*_NDM_. **(B)** An overview of the plasmid replicon types among the 171 *bla*_NDM_-harboring plasmids of *K. pneumoniae* analyzed using software PlasmidFinder.

Based on the results analyzed by ResFinder, 171 (3.84%) *bla*_NDM_-harboring plasmids (Supplementary Table S2) were identified from 4,451 plasmids of *K. pneumoniae*, which were downloaded from the GenBank Genome database. Among the 171 *bla*_NDM_-harboring plasmids of *K. pneumoniae*, nine different variants of *bla*_NDM_ were identified (Figure 1A). Among the nine variants of *bla*_NDM_, *bla*_NDM-1_ was found to be the predominant variant, accounting for 73.68% (126 *bla*_NDM-1_-harboring plasmids), followed by *bla*_NDM-5_, accounting for 16.37% (28 *bla*_NDM-5_-harboring plasmids) (Figure 1A).

### Replicon types of *bla*_NDM_-harboring plasmids of *Klebsiella pneumoniae*

Replicon typing of the 171 *bla*_NDM_-harboring plasmids of *K. pneumoniae* was executed using PlasmidFinder. Of the 171 plasmids, 154 were successfully identified with their replicon types, including 103 single-replicon plasmids and 51 multi-replicon plasmids (44 plasmids with two replicons, three plasmids with three replicons, and four plasmids with four replicons; Figure 1B; Supplementary Figure S1). For the 103 single-replicon plasmids harboring *bla*_NDM_ in *K. pneumoniae*, the TOP5 prevalent replicons (in descending order) were IncX3 (29 plasmids), IncC (15 plasmids), IncFIB(pQil) (11 plasmids), IncFII (11 plasmids), and IncFII(Yp) (11 plasmids). Of the 44 *bla*_NDM_-harboring plasmids with two replicons, 25 contained replicons IncFIB(pNDM-Mar) and IncHI1B(pNDM-MAR), which were the most prevalent two-replicon plasmids harboring *bla*_NDM_ in *K. pneumoniae* (Figure 1B; Supplementary Figure S1).

In summary, 21 of the 171 *bla*_NDM_-harboring plasmids of *K. pneumoniae* were found to carry the replicon of IncC, accounting for 12.28% of all the *bla*_NDM_-harboring plasmids of *K. pneumoniae* in this study (Figure 1B; Supplementary Figure S1). Notably, 91 of the 171 *bla*_NDM_-harboring plasmids in our study were found to be the IncF plasmids, including IncFI and IncFII plasmids, accounting for 53.22% of all the *bla*_NDM_-harboring plasmids of *K. pneumoniae* in this study (Figure 1B; Supplementary Figure S1).

### Diversity of the *bla*_NDM_-harboring plasmids in *Klebsiella pneumoniae*

**Figure 2:**
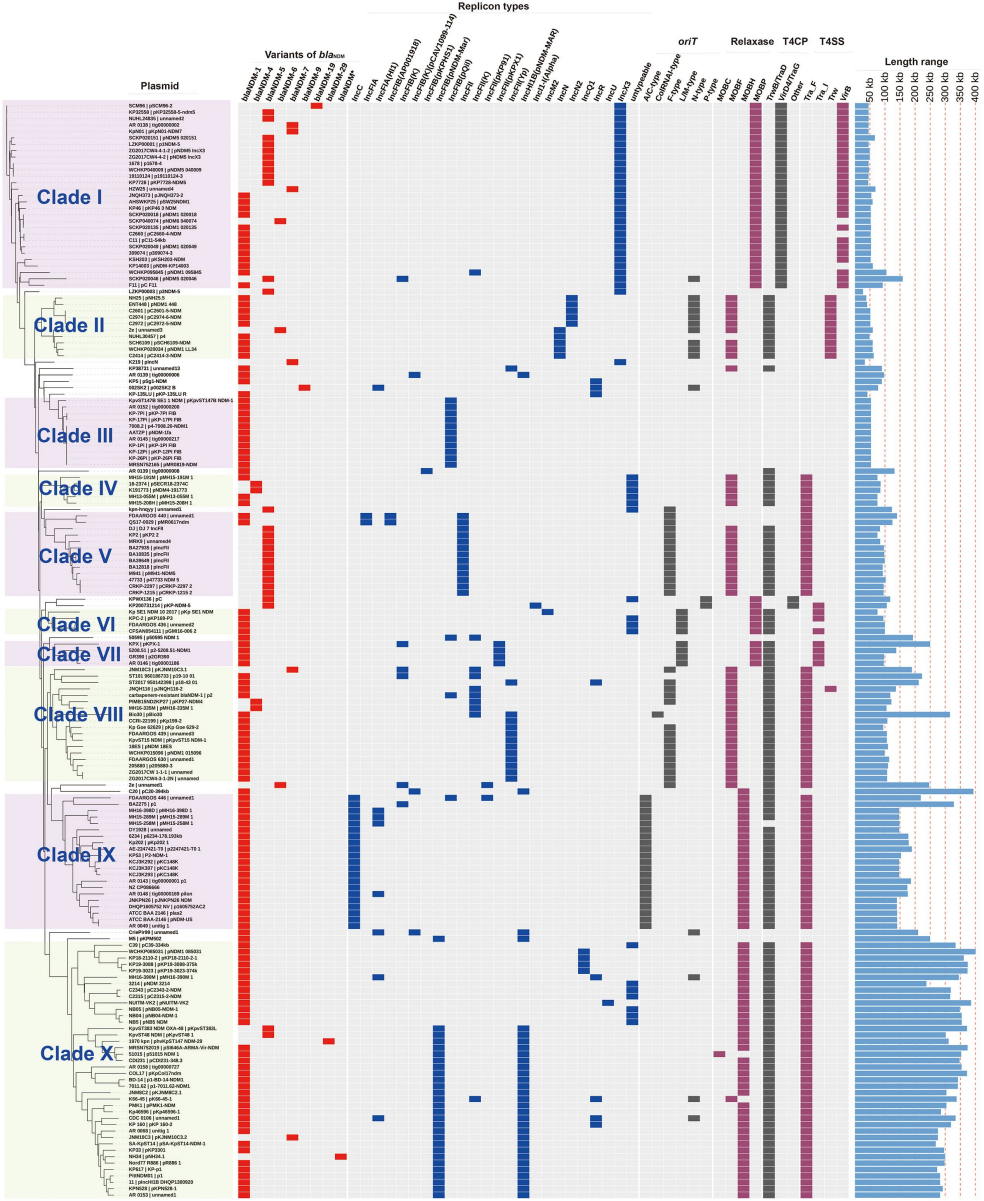
Details of variants of *bla*_NDM_ genes, replicon types, conjugative transfer regions, geographical distributions, host STs, and length distribution of the 171 *bla*_NDM_-harboring plasmids of *K. pneumoniae*. The four categories of information present in this figure include the phylogenetic cladogram, variants of *bla*_NDM_, replicon types, conjugative transfer regions (*oriT*, relaxase, T4CP, and T4SS), geographical distributions, ST types of host strains, and length distribution of the 171 *bla*_NDM_-harboring plasmids of *K. pneumoniae*. *bla*_NDM*_ representing an unknown variant of *bla*_NDM_.

To get the comprehensive overview of *bla*_NDM_-harboring plasmids in *K. pneumoniae*, we created a phylogenetic cladogram of the 171 *bla*_NDM_-harboring plasmids (Figure 2). Based on phylogenetic patterns of the 171 plasmids, combined with the replicon types, conjugative transfer regions, and genome sizes of the *bla*_NDM_-harboring plasmids, most of the 171 *bla*_NDM_-harboring plasmids were clustered into 10 main clades (clades I–X), representing 10 plasmid patterns carrying *bla*_NDM_ genes in *K. pneumoniae* (Table 1).

**Table 1:** Summary of the 171 *bla*_NDM_-harboring plasmids of *Klebsiella pneumoniae.*

Clade	Plasmid numbers	Main replicon types	Main *bla*_NDM_	Plasmid sizes (kb)	Main geographic distribution	Main ST types of hosts	Conjugative transfer region			
							*oriT*	Relaxase	T4CP	T4SS
I	29	IncX3	*bla*_NDM-1_	45.1–159.3	China	–	–	MOB_P_	VirD4/TraG	VirB-like
			*bla*_NDM-5_							
II	10	IncN	*bla*_NDM-1_	38.4–63.1	China	ST15	NW-type	MOB_F_	TrwB/TraD	Trw-like
III	11	IncFIB(pQil)	*bla*_NDM-1_	54	Italy, United States	ST147	–	–	–	–
IV	5	untypeable	*bla*_NDM-1_	75.3–86.0	Viet Nam	ST395, ST16	–	MOB_F_	TrwB/TraD	Tra_F-like
			*bla*_NDM-4_							
V	13	IncFII	*bla*_NDM-5_	75.3–140.6	India	ST16, ST147, ST2096	F-type	MOB_F_	TrwB/TraD	Tra_F-like
VI	4	untypeable	*bla*_NDM-1_	75.6–100.2	–	–	L/M-type	MOB_P_	TrwB/TraD	Tra_I-like
VII	4	IncFII(pKPX1)	*bla*_NDM-1_	96.8–250.4	–	–	L/M-type	MOB_P_	TrwB/TraD	Tra_I-like
VIII	18	IncF	*bla*_NDM-1_	94.4–316.2	–	–	F-type	MOB_F_	TrwB/TraD	Tra_F-like
IX	21	IncC	*bla*_NDM-1_	140.1–329.2	United States, China, Viet Nam	ST11, ST1967	A/C-type	MOB_H_	TrwB/TraD	Tra_F-like
X	40	IncF	*bla*_NDM-1_	238.0–401.6	China, United States	ST14, ST11, ST147	–	MOB_H_	TrwB/TraD	Tra_F-like

**Figure 3:**
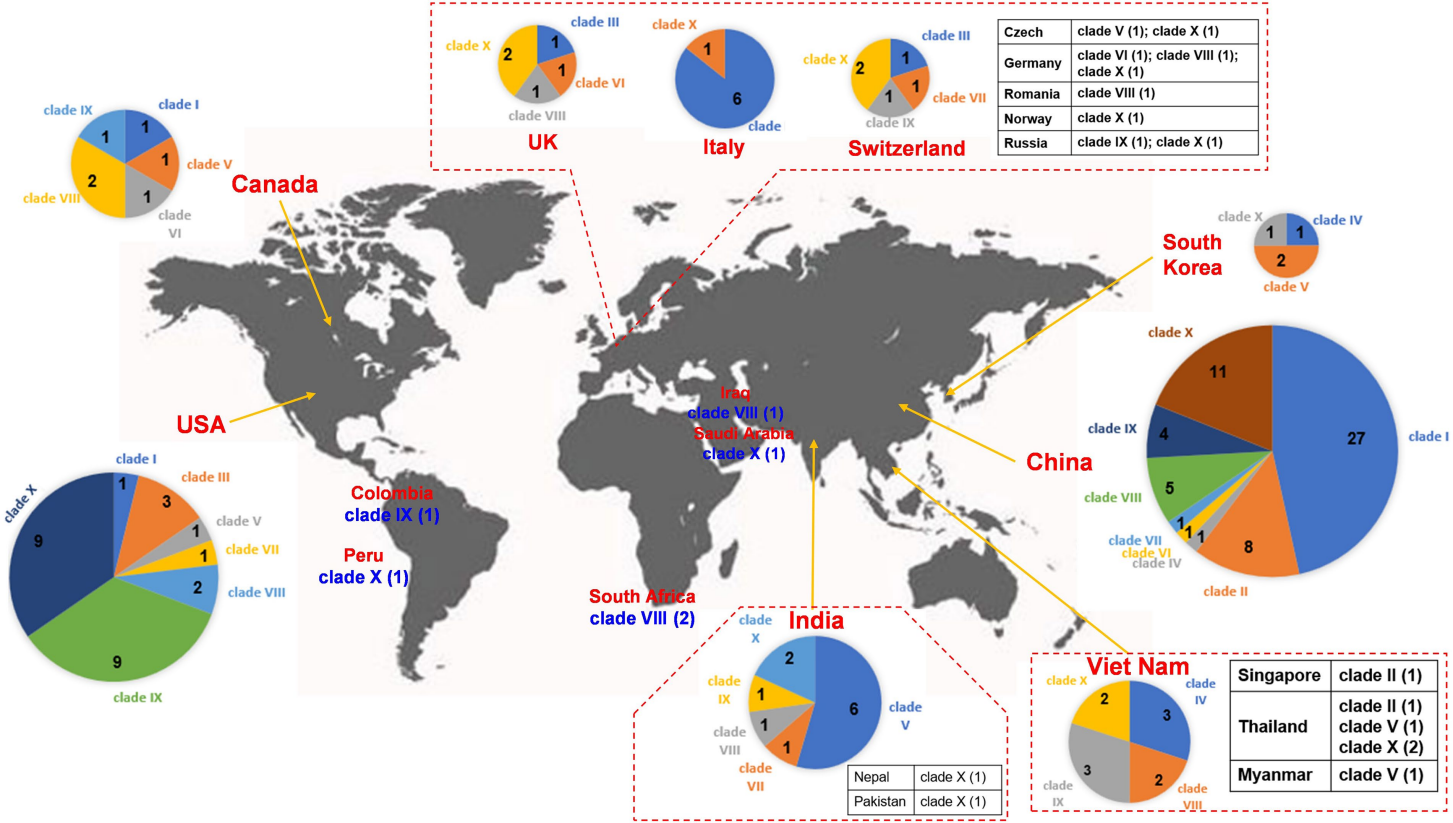
Worldwide distribution of *bla*_NDM_-harboring plasmids of *K. pneumoniae*. The geographical distribution of the 10 clades (Clade I–Clade X) from the *bla*_NDM_-positive plasmids of *K. pneumoniae* was calculated and displayed by pie chart. For the plasmids isolated in European countries, only those from the United Kingdom, Italy, and Switzerland were displayed by pie chart, others were displayed in the tabular form. For the plasmids isolated in Southeast Asia, only those from Viet Nam were displayed by pie chart, others were displayed in the tabular form. For the plasmids isolated in South Asia, only those from India were displayed by pie chart, others were displayed in the tabular form.

Clade I: A total of 29 IncX3 plasmids were found in the clade I cluster, mainly *bla*_NDM-1_ and *bla*_NDM-5_ (Figure 2). For the 29 IncX3 plasmids harboring *bla*_NDM_, their genome sizes varied from 45.1 to 159.3 kb (25th percentile = 46.2 kb; 75th percentile = 57.3 kb), with a median size of 53.1 kb (Supplementary Figure S2). For the conjugative transfer regions, all the plasmids belonging to clade I were found to carry genes encoding relaxases of the MOB_P_ family characterized by the domain “Relaxase (Pfam: PF03432),” T4CPs of the VirD4/TraG subfamily characterized by the domain “T4SS-DNA_transf (Pfam: PF02534),” and VirB-like T4SS gene clusters (Figure 2; Supplementary Figure S3). Members of clade I were mainly geographically distributed in China (Figure 3; Table 1; Supplementary Table S3). No predominant ST types of isolates were found in the plasmids harboring *bla*_NDM_ in *K. pneumoniae* (Table 1; Supplementary Table S3).

Clade II: Ten *bla*_NDM_-positive IncN plasmids were clustered into clade II, mainly carrying *bla*_NDM-1_ (Figure 2). The genome sizes of the 10 *bla*_NDM_-harboring IncN plasmids varied from 38.4 to 63.1 kb (25th percentile = 47.2 kb; 75th percentile = 59.8 kb), with a median size of 52.0 kb (Supplementary Figure S1). Almost all the IncN plasmids carried the NW-type *oriT*s and genes encoding relaxases of MOB_F_ family characterized by the domain “TrwC (PF08751).” All the 10 *bla*_NDM_-positive IncN plasmids carried the genes encoding T4CPs of the TrwB/TraD subfamily characterized by the domain “TrwB_AAD_bind (PF10412)” and Trw-like T4SS gene clusters (Figure 2; Supplementary Figure S4). The members of clade II were mainly geographically distributed in China (Figure 3; Table 1; Supplementary Table S3). The 10 *bla*_NDM_-positive IncN plasmids were distributed in seven ST types of *K. pneumoniae* strains, and four plasmids were distributed in *K. pneumoniae* ST15 (Table 1; Supplementary Table S3).

Clade III: Eleven *bla*_NDM-1_-positive IncF plasmids with the IncFIB(pQil) replicon were grouped into clade III, and most were 54-kb plasmids (Figure 2). Moreover, no conjugative transfer regions were identified in the 11 plasmids of clade III, indicating that the 11 plasmids should be non-transferable. Plasmids belonging to clade III were mainly geographically distributed in Italy and United States (Figure 3; Table 1; Supplementary Table S3). All the members of clade III were harbored by the strains of *K. pneumoniae* ST147 (Table 1; Supplementary Table S3).

Clade IV: Five *bla*_NDM_-positive untypeable plasmids were clustered into clade IV, involving three *bla*_NDM-1_-positive plasmids and two *bla*_NDM-4_-positive plasmids (Figure 2). These five untyped plasmids, with lengths ranging from 75.3 to 86.0 kb, all carried the genes encoding relaxases of MOB_F_ family, genes encoding T4CPs of TrwB/TraD subfamily, and Tra_F-like T4SS gene clusters (Figure 2; Supplementary Figure S5). For the five plasmids, three were found in Viet Nam, one was found in China, and one was found in South Korea (Figure 3; Table 1; Supplementary Table S3). The STs of *K. pneumoniae* host strains containing the clade IV plasmids were distributed into ST395 and ST16 (Table 1; Supplementary Table S3).

Clade V: Thirteen plasmids with the IncFII replicon, mainly carrying *bla*_NDM-5_, were classified into the clade V (Figure 2). For the 13 IncFII plasmids harboring *bla*_NDM_, genome sizes varied from 75.3 to 140.6 kb (25th percentile = 88.8 kb; 75th percentile = 101.4 kb), with a median size of 96.2 kb (Supplementary Figure S1). They all carried the F-type *oriT*s and Tra_F-like T4SS gene clusters (Figure 2; Supplementary Figure S6). Most of the plasmids clustered into clade V were found to carry genes encoding relaxases of the MOB_F_ family and genes encoding T4CPs of the TrwB/TraD subfamily (Figure 2). The members of clade V were widely distributed in India, Southeast Asia, North America, East Asia, and Europe, with the highest prevalence in India (Figure 3; Table 1; Supplementary Table S3). The STs of *K. pneumoniae* host strains containing all Clade V plasmids were mainly distributed in ST16, ST147, and ST2096 (Table 1; Supplementary Table S3).

Clade VI: Four *bla*_NDM-1_-positive plasmids, including one IncM2 plasmid and three untyped plasmids, were classified into a small cluster named clade VI in our study (Figure 2). These four plasmids, with lengths ranging from 75.6 to 100.2 kb, all carried the L/M-type *oriT*s, genes encoding relaxases of MOB_P_ family, genes encoding T4CPs of TrwB/TraD subfamily, and Tra_I-like T4SS gene clusters (Figure 2; Supplementary Figure S7). The four plasmids were sporadically discovered in Canada, Germany, United Kingdom, and China (Figure 3; Table 1; Supplementary Table S3). No prevalent STs of *K. pneumoniae* host strains containing all clade VI plasmids were found (Table 1; Supplementary Table S3).

Clade VII: Four *bla*_NDM-1_-positive plasmids with the IncFII(pKPX1) replicon were classified into clade VII (Figure 2). The genome sizes of the four IncFII(pKPX1) plasmids varied from 96.8 to 250.4 kb. Similar to the conjugative transfer regions of plasmids belonging to clade VI, they all carried the L/M-type *oriT*s, genes encoding relaxases of the MOB_P_ family, genes encoding T4CPs of the TrwB/TraD subfamily, and Tra_I-like T4SS gene clusters (Figure 2; Supplementary Figure S8). The four plasmids were sporadically discovered in United States, India, Switzerland, and China (Figure 3; Table 1; Supplementary Table S3). No obvious common STs of strains were found (Table 1; Supplementary Table S3).

Clade VIII: Eighteen IncF plasmids, mainly carrying *bla*_NDM-1_, were grouped into the clade VIII cluster (Figure 2). Most of the IncF plasmids contained the IncFII(Yp) or IncFII(K) replicon in their genomes. For the 18 *bla*_NDM_-harboring plasmids of clade VIII, genome sizes varied from 94.4 to 316.2 kb (25th percentile = 106.8 kb, 75th percentile = 150.1 kb), with a median size of 110.6 kb (Supplementary Figure S1). Most of the plasmids of clade VIII were found to contain the F-type *oriT*s. They all carried the genes encoding relaxases of the MOB_F_ family, genes encoding T4CPs of the TrwB/TraD subfamily, and Tra_F-like T4SS gene clusters (Figure 2; Supplementary Figure S9). Notably, *K. pneumoniae* strain JNQH116 plasmid pJNQH116-2 (NZ_CP070900), belonging to the clade VIII cluster, was found to contain both Tra_F-like and Trw-like T4SS gene clusters in its genome. For clade VIII, its members were widely geographically distributed all over the world, including China, India, Southeast Asia, Middle East, North America (Canada and United States), South Africa, and some European countries (e.g., Germany, Romania, and the United Kingdom; Figure 3; Table 1; Supplementary Table S3). No prevalent STs of *K. pneumoniae* host strains containing all clade VIII plasmids were found (Table 1; Supplementary Table S3).

Clade IX: A total of 21 IncC plasmids carrying *bla*_NDM-1_ were grouped into the clade IX cluster of the phylogenetic cladogram (Figure 2). Their genome sizes varied from 140.1 to 329.2 kb, with the 25th percentile, median size, and 75th percentile being 144.3, 147.9, and 178.7 kb, respectively (Supplementary Figure S1). For the conjugative transfer modules, all the plasmids belonging to clade IX carried the A/C-type *oriT*s, genes encoding relaxases of the MOB_H_ family characterized by the domain “TraI_2 (Pfam: PF07514),” mostly genes encoding T4CPs of TrwB/TraD subfamily, and Tra_F-like T4SS gene clusters (Figure 2; Supplementary Figure S10). The IncC plasmids harboring *bla*_NDM-1_ were widely geographically distributed all over the world, mainly in the United States, Viet Nam, and China (Figure 3; Table 1; Supplementary Table S3). ST11 and ST1967 were the common STs strains containing *bla*_NDM-1-_harboring IncC plasmids (Table 1; Supplementary Table S3).

Clade X: A total of 40 mega plasmids, where the length range varied from 238.0 to 401.6 kb (25th percentile = 293.5 kb; median = 327.3 kb; 75th percentile = 355.1 kb), mainly carrying *bla*_NDM-1_, were grouped into a large cluster, named clade X in our study (Figure 2). Of the plasmids belonging to clade X, 27 (67.5%) were found to contain both replicons IncFIB(pNDM-Mar) and IncHI1B(pNDM-MAR), seven (17.5%) were unable to be typed, and four (10.0%) were IncQ1 plasmids. Moreover, all the plasmids of clade X carried genes encoding T4CPs of the TrwB/TraD subfamily and Tra_F-like T4SS gene clusters (Figure 2; Supplementary Figure S11). Most of the plasmids belonging to clade X were found to have no *oriT* and harbored the genes encoding relaxases of the MOB_H_ family. For the clade with the largest number, clade X, its members were widely distributed all over the world, mainly in China and the United States (Figure 3; Table 1; Supplementary Table S3). ST14, ST11, and ST147 were the common STs strains containing the plasmids of clade X (Table 1; Supplementary Table S3).

**Figure 4:**
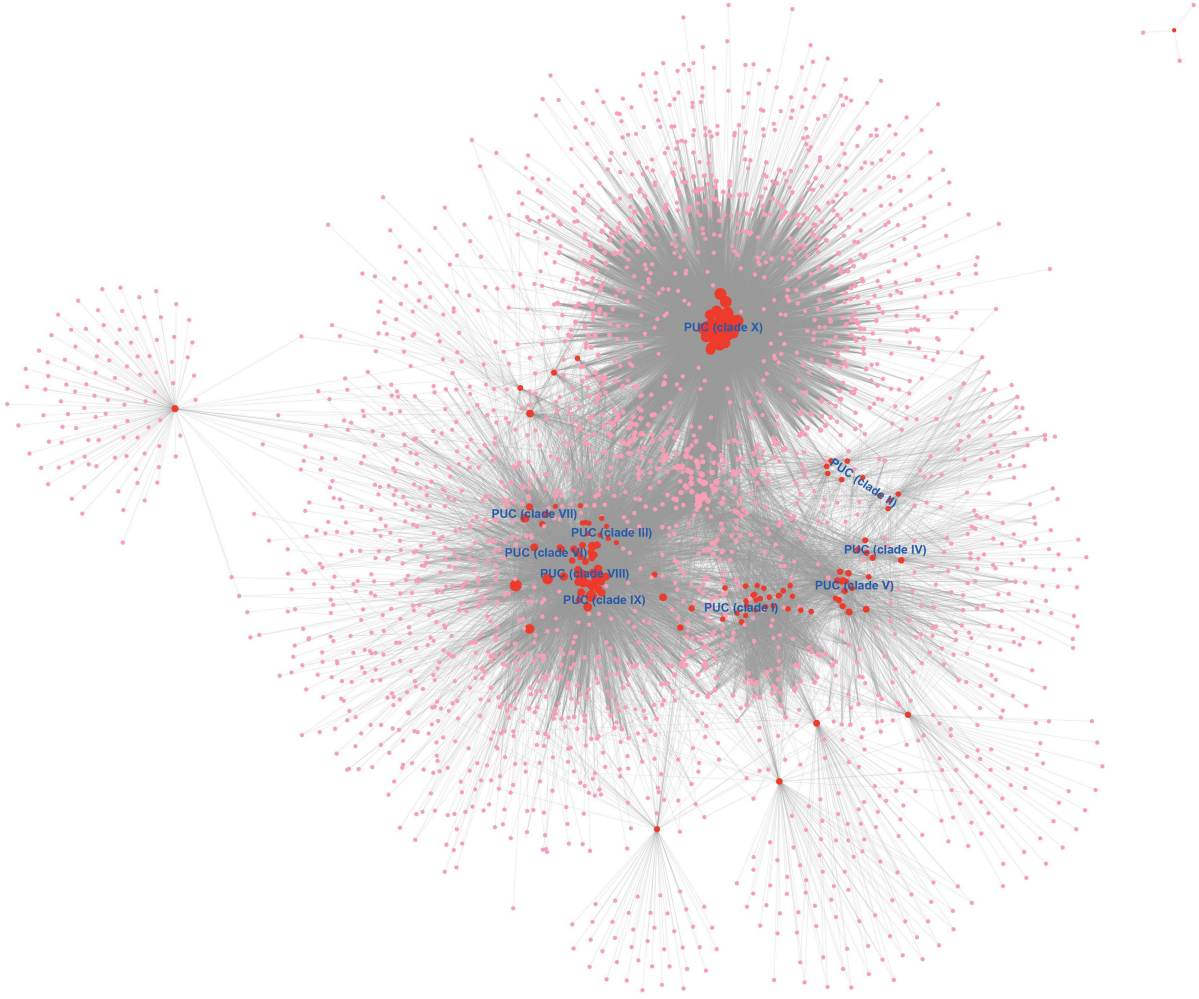
The PU-HPC bipartite network visualization of the *bla*_NDM_-harboring plasmids of *K. pneumoniae*. Plasmid units (PUs, 171) and homologous protein clusters (HPCs, 2,502) are represented as colored circles, with red for PUs and pink for HPCs. The size of a circle is ranked as the degree calculated with the Cytoscape’s built-in NetworkAnalyzer tool. The 10 clades (Clade I–Clade X) were labeled into the location of corresponding PUCs.

We also perform a bipartite network analysis with the 171 *bla*_NDM_-harboring plasmids of *K. pneumoniae.* The bipartite network consisted of two classes of nodes: 171 plasmid units (PUs) and 2,502 homologous protein clusters (HPCs, protein families according to amino acid sequence identity, coverage, and *E*-value; Figure 4). Edges connected every PU with the HPC that it contained. The PUs of the bipartite network clearly showed distinct clustering phenomena. Overall, one homologous PU clusters (PUCs) contained the almost the same members of Clade X in the analysis above (Figure 4), which was clearly distinct from other PUs. One large region including clades III, VI – IX was also identified, which were mostly IncF and IncC plasmids. In addition, clades I, II, IV, and V were also found their corresponding PUCs in the PU-HPC bipartite network.

## Discussion

Global spread of the NDM-type carbapenemases can be partly attributed to the dissemination of various *bla*_NDM_-harboring plasmids (Lee et al., 2016; Dong et al., 2022). Therefore, to characterize plasmids harboring *bla*_NDM_ in *K. pneumoniae*, we systematically analyzed the variants of *bla*_NDM_, replicon types, and conjugative transfer regions of 4,451 plasmids belonging to *K. pneumoniae* from the NCBI GenBank database. Overall, 171 *bla*_NDM_-harboring plasmids of *K. pneumoniae* were identified.

In our study, nine different variants of *bla*_NDM_ were identified from the 171 *bla*_NDM_-harboring plasmids in *K. pneumoniae*, with *bla*_NDM-1_ and *bla*_NDM-5_ being highly prevalent; *bla*_NDM-1_-carrying plasmids were the most prevalent and accounted for 73.68% of the 171 *bla*_NDM_-harboring plasmids. NDM-1 was first reported in 2008 on a 180-kb plasmid of *K. pneumoniae* strain isolated from a Swedish patient hospitalized in New Delhi, India (Yong et al., 2009). After the first report, NDM-1 was reported in many clinical isolates, mainly *K. pneumoniae* and *E. coli* (Kumarasamy et al., 2010). In addition, *bla*_NDM-5_ was another common variant in our work, accounting for 16.37% of the 171 *bla*_NDM_-harboring plasmids. The variant NDM-5 was first reported on an IncF plasmid of *E. coli* EC405, isolated from a 41-year-old British patient who had a travel history to India (Hornsey et al., 2011). Notably, *bla*_NDM-5_ was reported to be the predominant variant in *bla*_NDM_-harboring plasmids of *E. coli* (Zhang et al., 2021).

Our results showed that IncX3 single-replicon plasmids were important carriers of *bla*_NDM_ in *K. pneumoniae*, mainly *bla*_NDM-1_ and *bla*_NDM-5_. IncX3 plasmid is narrow-host range plasmids in Enterobacterales (Johnson et al., 2012), which has been reported to harbor diverse carbapenemase genes in CRE worldwide (Mouftah et al., 2019). Of the 29 *bla*_NDM_-harboring IncX3 plasmids grouped into clade I, most were relatively small with lengths of 46–57 kb (25th percentile = 46.2 kb; median size = 53.1 kb; 75th percentile = 57.3 kb). Based on the results analyzed by the oriTfinder software, all the 29 *bla*_NDM_-harboring IncX3 plasmids of clade I contained genes encoding for relaxases belonging to the MOB_P_ family, with TraI protein encoded by the IncPα plasmid RP4 (Pansegrau et al., 1993) as a representative. T4CPs encoded by the 29 *bla*_NDM_-harboring IncX3 plasmids of clade I belonged to the VirD4/TraG subfamily, with the TraG protein of plasmid RP4 and the VirD4 protein of Ti plasmids as representatives (Gomis-Rüth et al., 2004). The *bla*_NDM_-harboring IncX3 plasmids classified into clade I contained VirB-like T4SS gene clusters, which are the best-characterized T4SS (Guglielmini et al., 2014). However, no known *oriT* site was found in most of the IncX3 plasmids harboring *bla*_NDM_ belonging to clade I of the phylogenetic cladogram, indicating a new type of *oriT* site, different from the nine *oriT* families collected in the oriTDB database (Li et al., 2018).

We found 10 *bla*_NDM_-harboring IncN plasmids, with IncN or IncN2 replicons, clustered into clade II of the phylogenetic cladogram. They were also relatively small plasmids, with genome sizes varying from 38.38 to 63.05 kb. These *bla*_NDM_-harboring IncN plasmids carried the NW-type *oriT*s, which were characterized by the conserved nick region KGTST|ATAGC (“|” refers to the *nic* site of *oriT*), with *oriT* sites of IncN plasmid R46 (Hall and Vockler, 1987) and IncW plasmid R388 (Revilla et al., 2008) as representatives. Almost all the plasmids of clade II contained genes coding for relaxases of the MOB_F_ family, which was characterized by the domain “TrwC (PF08751),” with R388 TrwC and F TraI as representatives (de la Cruz et al., 2010). The T4CPs encoded by the *bla*_NDM_-positive IncN plasmids belonged to the TrwB/TraD subfamily, which was characterized by the domain “TrwB_AAD_bind (PF10412),” with the TrwB encoded by plasmid R388 from *E. coli* as a representative (Gomis-Rüth et al., 2004). In addition, the *bla*_NDM_-positive IncN plasmids carried Trw-like T4SS gene clusters. The Trw T4SS clusters were regarded as the bacterial conjugation machines that mediate the spread of plasmids among bacterial populations (e.g., the trw locus of broad-host-range IncW plasmid R388; Seubert et al., 2003) while also mediating host-specific erythrocyte infection (e.g., the pathogenesis-related Trw system of Bartonella; Vayssier-Taussat et al., 2010).

Our work showed that 21 IncC plasmids carrying *bla*_NDM-1_, with genome sizes from 140.1 kb to 329.2 kb, were clustered into the clade IX of the phylogenetic cladogram constructed by the 171 *bla*_NDM_-harboring plasmids in *K. pneumoniae*. The broad-host-range IncC mega plasmids are essential contributors to the dissemination of antibiotic resistance genes, and more than 200 fully sequenced IncC plasmids have been reported (Ambrose et al., 2018). The *bla*_NDM-1_-harboring IncC plasmids of clade IX contained the A/C-type *oriT*s, with the *oriT* site of IncA/C conjugative pVCR94ΔX from Vibrio cholera as the prototype (Carraro et al., 2014). Furthermore, these *bla*_NDM-1_-harboring IncC plasmids carried genes encoding relaxases of the MOB_H_ family, characterized by the domain “TraI_2 (Pfam: PF07514),” with TraI encoded by IncHI plasmid R27, TraI encoded by IncA/C plasmid pIP1202, TraI encoded by IncJ plasmid R391, and TraI encoded by IncT plasmid Rts1 as representatives (de la Cruz et al., 2010). In addition, most of the IncC plasmids clustered into clade IX contained genes encoding T4CPs of the TrwB/TraD subfamily and Tra_F-like T4SS gene clusters.

In our work, 53.22% (91 out of 171 plasmids) of the *bla*_NDM_-harboring plasmids of *K. pneumoniae* were found to be IncF plasmids, and most were multi-replicon IncF plasmids, especially IncFI-type plasmids. IncF plasmids are commonly low-copy-number plasmids, >100 kb in size (Villa et al., 2010); however, in our study, the *bla*_NDM_-harboring IncF plasmids in *K. pneumoniae* were heterogeneous in size. For example, the *bla*_NDM-1_-positive IncF plasmids, with the IncFIB(pQil) replicon, clustered into clade III were mostly 54-kb plasmids; the genome sizes of the IncFII plasmids grouped into clade V varied from 75.31 to 140.6 kb (25th percentile = 88.81 kb; 75th percentile = 101.4 kb); and the 27 plasmids with replicon IncFIB(pNDM-Mar) belonging to clade X were > 250 kb in size. The IncF plasmids comprise a diverse set of conjugative plasmids frequently found in Enterobacterales, which contribute to spreading AMR genes (Villa et al., 2010; Carattoli, 2011). The *bla*_NDM_-harboring IncF plasmids in *K. pneumoniae* were also heterogeneous in types of conjugative transfer regions. The IncFII-type plasmids, including clades V and VIII, carried F-type *oriT*s, genes encoding relaxases of the MOB_F_ family, genes encoding T4CPs of the TrwB/TraD subfamily, and Tra_F-like T4SS gene clusters belonging to the classical F-like conjugative system (de la Cruz et al., 2010). Mega plasmids with replicons IncFIB(pNDM-Mar):IncHI1B(pNDM-MAR) belonging to clade X mostly harbored the genes encoding relaxases of the MOB_H_ family. In our study, we found 11 *bla*_NDM-1_-positive IncFIB(pQil) plasmids classified into clade III without any classical conjugative transfer regions, which were predicted as non-transferable plasmids.

## Conclusion

In this study, we analyzed the variants of *bla*_NDM_, replicon types, conjugative transfer regions, host STs, and geographical distributions of 171 *bla*_NDM_-harboring plasmids from 4,451 *K. pneumoniae* plasmids, which were downloaded from the GenBank database. Nine variants of *bla*_NDM_ were found among the 171 *bla*_NDM_-positive plasmids, with *bla*_NDM-1_ (73.68%) and *bla*_NDM-5_ (16.37%) as the most dominant. Over half of the *bla*_NDM_-harboring plasmids of *K. pneumoniae* were classified into IncF plasmids. In addition, IncX3 single-replicon plasmids (46–57 kb), IncN plasmids (38.4–63.1 kb), IncC plasmids (140.1–329.2 kb) were also the common carriers of *bla*_NDM_ in *K. pneumoniae*. The *bla*_NDM_-harboring IncX3 and IncN plasmids were mainly geographically distributed in China. The IncC plasmids harboring *bla*_NDM-1_ were widely geographically distributed all over the world, mainly in the United States, China, and Viet Nam. This study provides important insights into the diversity of *bla*_NDM_-harboring plasmids in *K. pneumoniae* and further addresses their role in the acquisition and spread of resistance genes. However, the genetic diversity and characteristics of *bla*_NDM_-harboring plasmids in other Gram-negative species need further study in the future.

## Data availability statement

The datasets presented in this study can be found in online repositories. The names of the repository/repositories and accession number(s) can be found in the article/Supplementary material.

## Author contributions

XL, LuL, and XD: conceptualization. ZhuZ: methodology. LeL and LiL: software. SH and JY: validation. WL, LZ, QL, and ZhiZ: formal analysis. ZhuZ and XL: writing—original draft preparation. XL and LuL: writing—review and editing. XL: supervision. All authors contributed to the article and approved the submitted version.

## Funding

This work was supported financially by the grants from the National Natural Science Foundation of China (grant nos. 81902460 and 82002170), the Xiangshan Talent Project of Zhuhai People’s Hospital (grant no. 2020XSYC-02), and the Cultivation Project of Zhuhai People’s Hospital (2019PY-19).

## Conflict of interest

The authors declare that the research was conducted in the absence of any commercial or financial relationships that could be construed as a potential conflict of interest.

## Publisher’s note

All claims expressed in this article are solely those of the authors and do not necessarily represent those of their affiliated organizations, or those of the publisher, the editors and the reviewers. Any product that may be evaluated in this article, or claim that may be made by its manufacturer, is not guaranteed or endorsed by the publisher.

## Footnotes

^1^ https://bioinfo-mml.sjtu.edu.cn/oriTDB/browse_oriT_type_p.php

^2^ https://bioinfo-mml.sjtu.edu.cn/oriTDB/browse_relaxase.php

^3^ https://bioinfo-mml.sjtu.edu.cn/SecReT4/browse_type.php

^4^ https://bioinfo-mml.sjtu.edu.cn/oriTDB/browse_t4cp.php

^5^ https://www.ncbi.nlm.nih.gov/genome/browse/#!/plasmids/815/

^6^ https://card.mcmaster.ca

^7^ https://www.ncbi.nlm.nih.gov/genome/browse/#!/prokaryotes/815/

^8^ https://cge.food.dtu.dk/services/MLST/

^9^ https://bioinfo-mml.sjtu.edu.cn/oriTDB/index.php

^10^ https://bioinfo-mml.sjtu.edu.cn/SecReT4/
