# Supplementary material for: In silico characterization of blaNDM-harboring plasmids in Klebsiella pneumoniae
Source: Front Microbiol. 2022 Nov 23;13:1008905. doi: 10.3389/fmicb.2022.1008905 (PMC9727287; doi:10.3389/fmicb.2022.1008905)
Supplement: Supplementary file 1 [file Data_Sheet_1.DOCX]

*In Silico* Characterization of *bla*_NDM_-Harboring Plasmids in *Klebsiella pneumoniae*

**Genome sizes of *bla*_NDM_-harboring plasmids of *K. pneumoniae***

We analyzed the genome sizes of the 171 *bla*_NDM_-harboring plasmids of *K. pneumoniae* and the sizes of plasmids harboring *bla*_NDM-1_ and *bla*_NDM-5_. Among the 171 *bla*_NDM_-harboring plasmids, genome sizes varied from 27.7 kb to 401.6 kb, with the 25th percentile, median size, and 75th percentile being 59.4 kb, 107.6 kb, and 250.4 kb, respectively (Fig. *). For the 126 *bla*_NDM-1_-harboring plasmids, genome sizes varied from 38.4 kb to 401.6 kb, with the 25th percentile, median size, and 75th percentile being 62.3 kb, 140.4 kb, and 284.1 kb, respectively (Fig. 1B). For the 28 plasmids harboring *bla*_NDM-5_, genome sizes varied from 27.7 kb to 372.8 kb, with the 25th percentile, median size, and 75th percentile being 46.5 kb, 88.8 kb, and 102.2 kb, respectively.

Box plot of the length distribution of the 28 *bla*_NDM-5_-harboring plasmids, 126 *bla*_NDM-1_-harboring plasmids, and all the 171 *bla*_NDM_-harboring plasmids of *K. pneumoniae*.

**Table legends：**

**Table S1.** Information of 4451 plasmids of *K. pneumoniae* included in this study.

**Table S2.** Details of *bla*_NDM_-harboring plasmids of *K. pneumoniae* identified by the software ResFinder.

**Table S3.** Clinical data of the 171 plasmids in *K. pneumoniae.*

**Figure legends:**

**Figure S1**. Histogram of number of combination modes of different replicons among the 171 *bla*_NDM_-harboring plasmids of *K. pneumoniae* analyzed using software PlasmidFinder.

**Figure S2**. Length distribution of the *bla*_NDM_-harboring plasmids in different clades (clades I–X).

**Figure S3**. Details of the VirB-like T4SS of plasmid pSCM96-2 in *K. pneumoniae* strain SCM96 (NZ_CP028718) and other 28 IncX3 plasmids harboring *bla*_NDM_ clustered into clade I.

**Figure S4**. Details of the Trw-like T4SS of *K. pneumoniae* strain 2e plasmid unnamed3 (CP040178) and other nine IncN plasmids harboring *bla*_NDM_ clustered into clade II.

**Figure S5**. Details of the Tra_F-like T4SS of plasmid pNDM4-191773 in *K. pneumoniae* strain K191773 (NZ_CP080366) and other four plasmids harboring *bla*_NDM_ clustered into clade IV.

**Figure S6**. Trw-like T4SS of *K. pneumoniae* strain CRKP-1215 plasmid pCRKP-1215_2 (NZ_CP024840) and other 12 IncFII plasmids clustered into clade V.

**Figure S7**. Details of the Tra_I-like T4SS of plasmids harboring *bla*_NDM_ clustered into clade VI. (A) Tra_I-like T4SS of the three untyped plasmids. (B) Tra_I-like T4SS of the IncM2 plasmid pKp_SE1_NDM in *K. pneumoniae* strain Kp_SE1_NDM_10_2017 (NZ_CM010662).

**Figure S8**. Tra_I-like T4SS gene clusters of plasmid pKPX-1 in *K. pneumoniae* strain KPX (AP012055) and other three plasmids harboring *bla*_NDM_ clustered into clade VII.

**Figure S9**. Trw-like T4SS of *K. pneumoniae* strain JNQH116 plasmid pJNQH116-2 (NZ_CP070900) and other 17 IncF plasmids harboring *bla*_NDM_ clustered into clade VIII.

**Figure S10**. Details of the Tra_F-like T4SS of plasmid p6234-178.193kb in *K. pneumoniae* strain 6234 (NZ_CP010391) and other 20 IncC plasmids harboring *bla*_NDM_ clustered into clade IX.

**Figure S11**. Details of the Tra_F-like T4SS of *K. pneumoniae* strain K66-45 plasmid pK66-45-1 (NZ_CP020902) and other 39 plasmids harboring *bla*_NDM_ clustered into clade X.


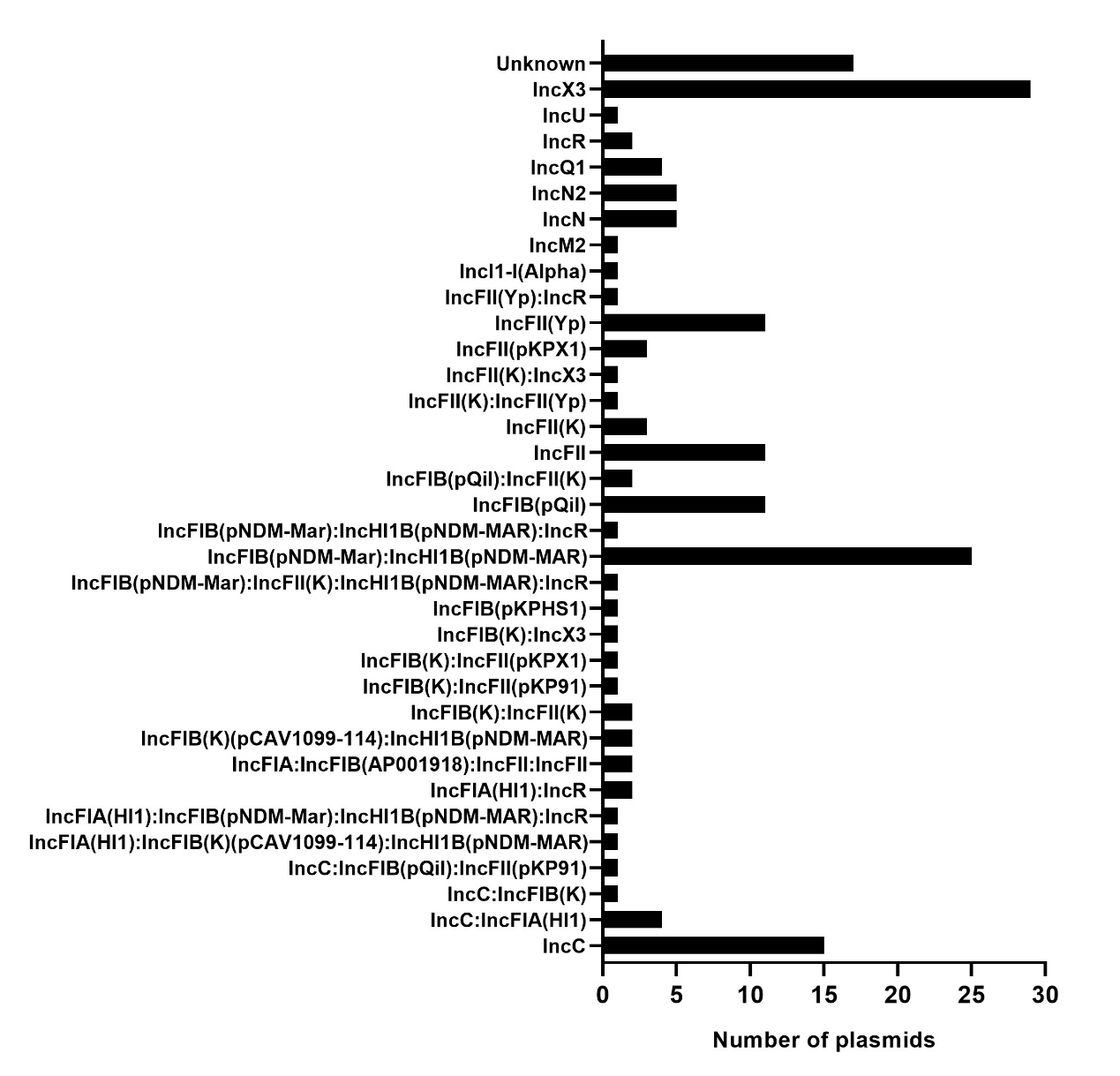
 Figure S1. Histogram of number of combination modes of different replicons among the 171 *bla*_NDM_-harboring plasmids of *K. pneumoniae* analyzed using software PlasmidFinder.

Figure S2. Length distribution of the *bla*_NDM_-harboring plasmids in different clades (clades I–X).


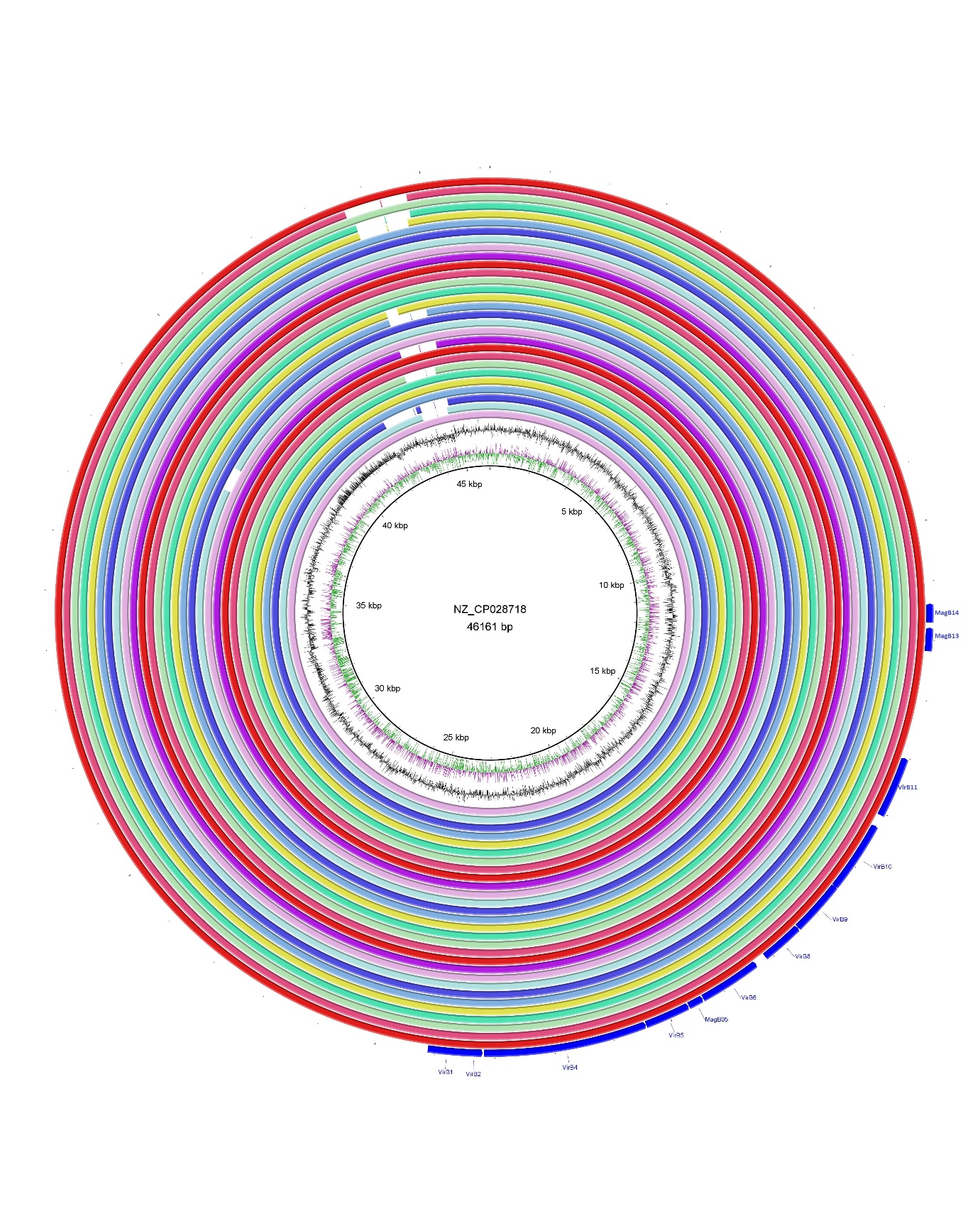


Figure S3. Details of the VirB-like T4SS of plasmid pSCM96-2 in *K. pneumoniae* strain SCM96 (NZ_CP028718) and other 28 IncX3 plasmids harboring *bla*_NDM_ clustered into clade I.


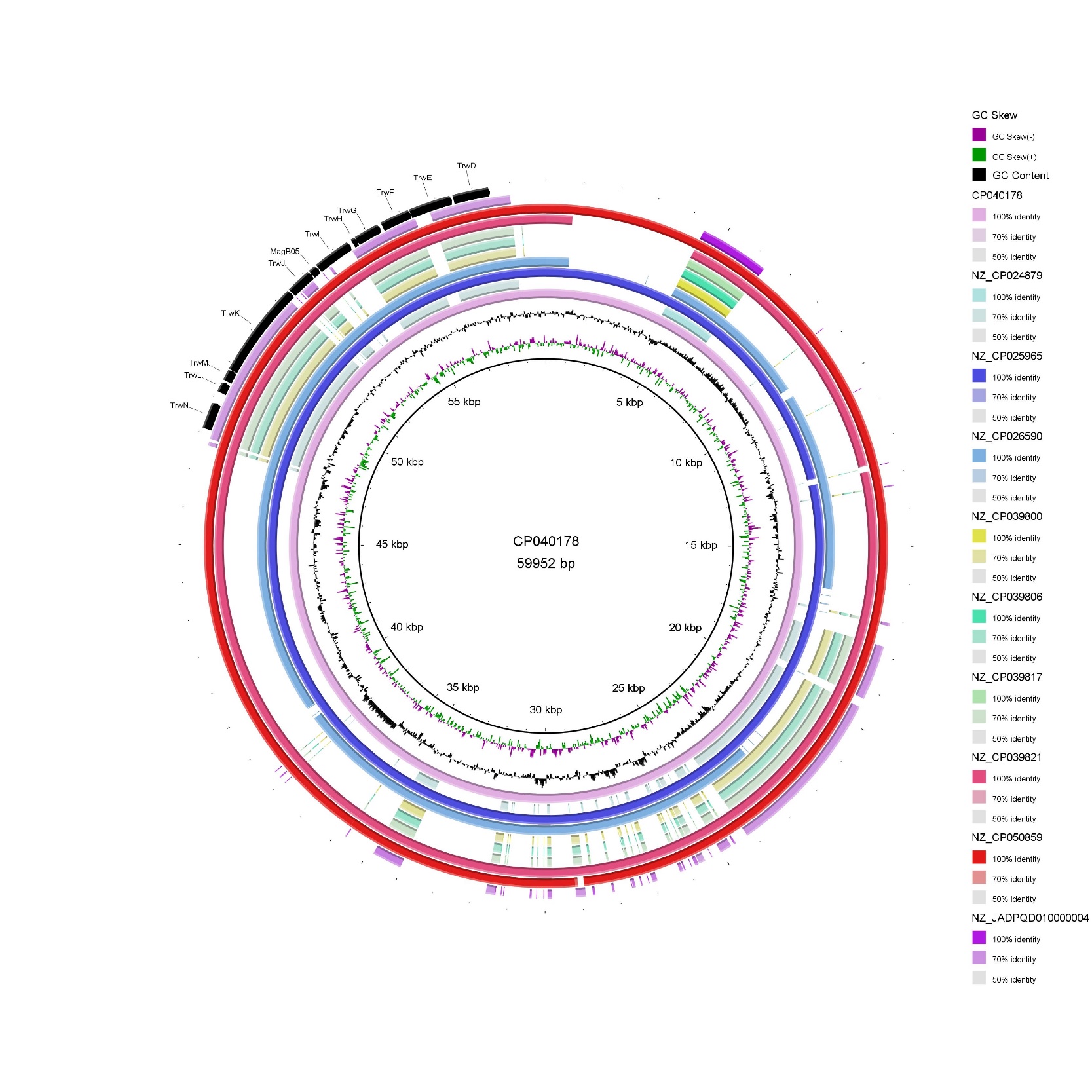


Figure S4. Details of the Trw-like T4SS of *K. pneumoniae* strain 2e plasmid unnamed3 (CP040178) and other nine IncN plasmids harboring *bla*_NDM_ clustered into clade II.


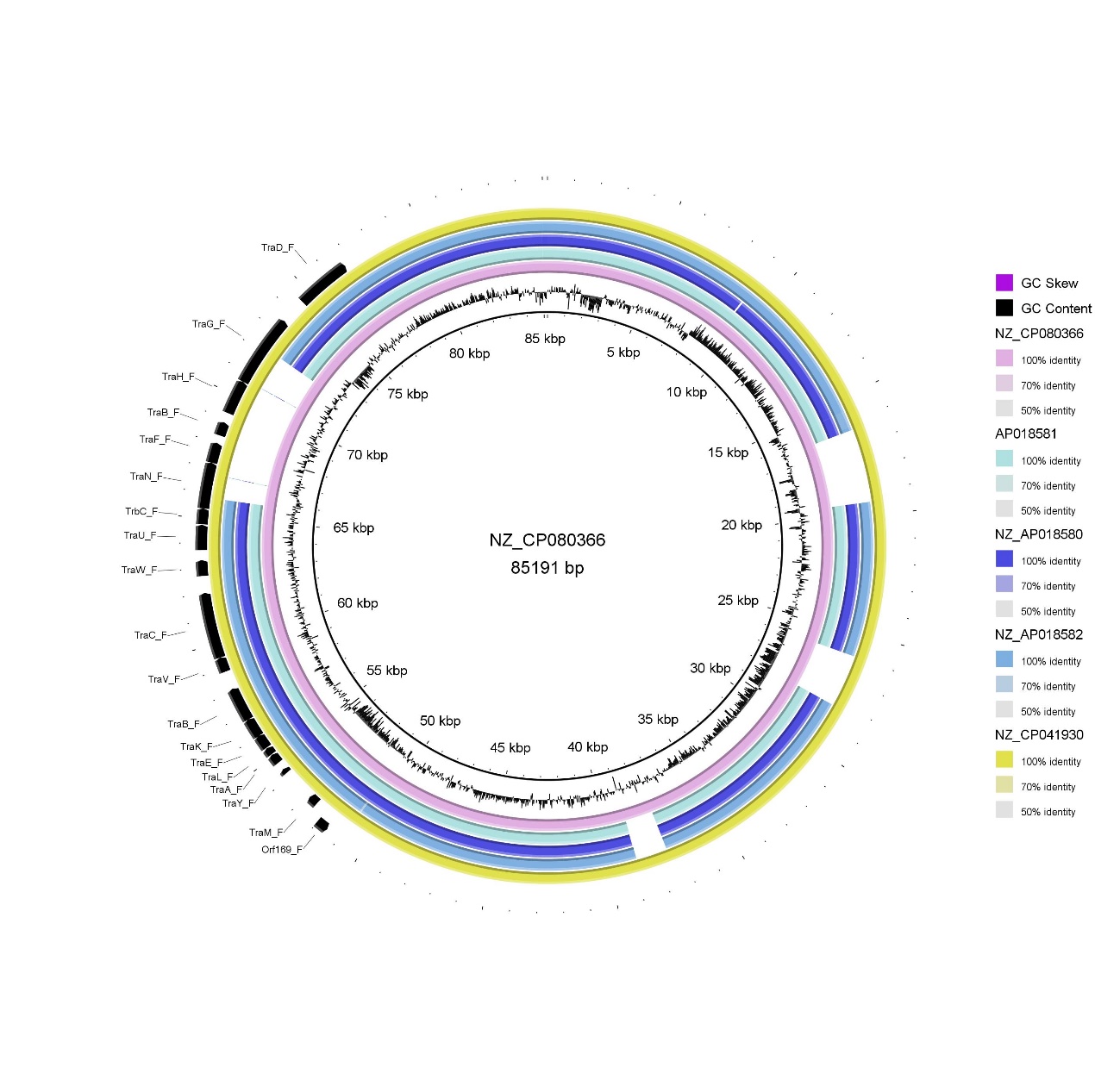


Figure S5. Details of the Tra_F-like T4SS of plasmid pNDM4-191773 in *K. pneumoniae* strain K191773 (NZ_CP080366) and other four plasmids harboring *bla*_NDM_ clustered into clade IV.


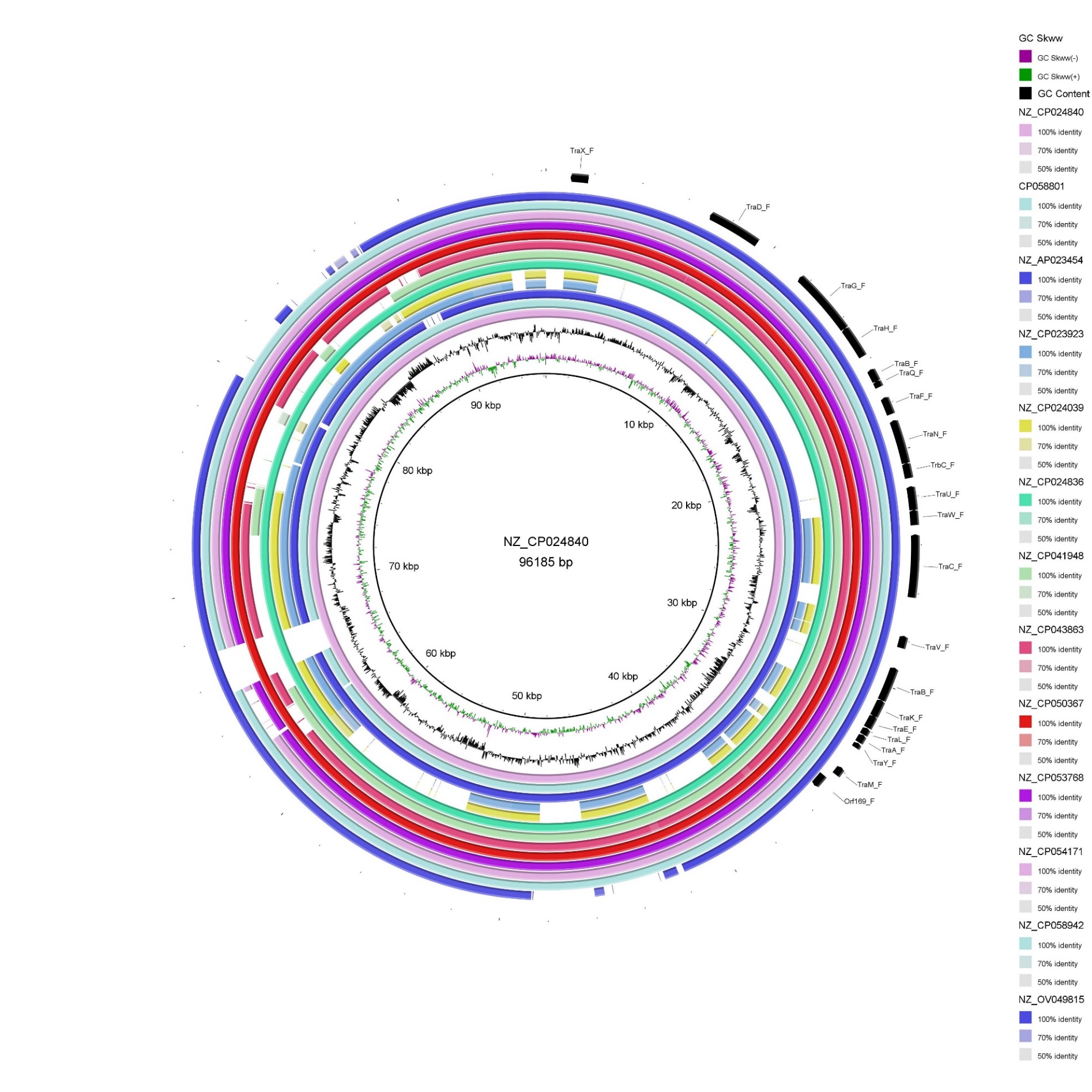


Figure S6. Trw-like T4SS of *K. pneumoniae* strain CRKP-1215 plasmid pCRKP-1215_2 (NZ_CP024840) and other 12 IncFII plasmids clustered into clade V.


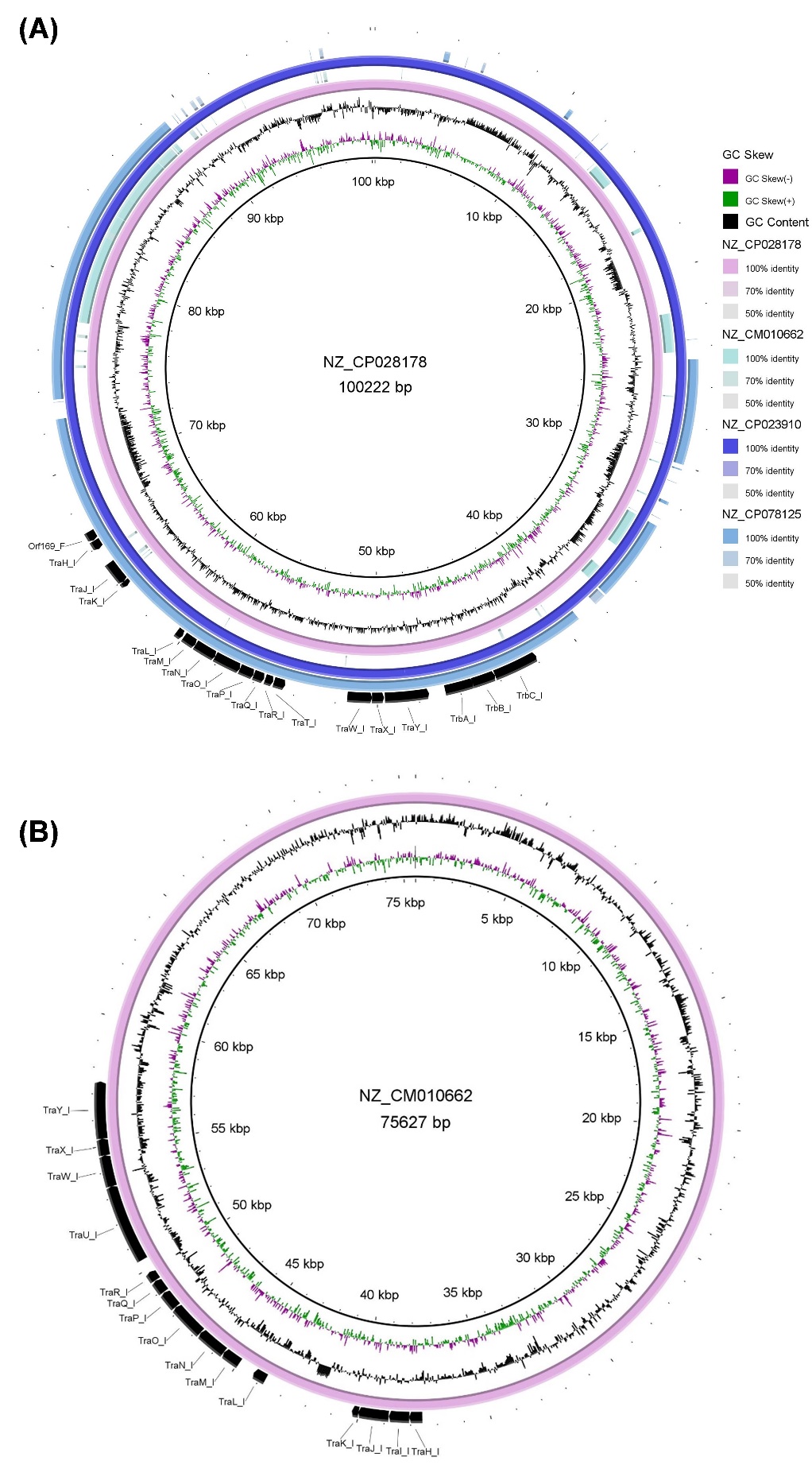


Figure S7. Details of the Tra_I-like T4SS of plasmids harboring *bla*_NDM_ clustered into clade VI. (A) Tra_I-like T4SS of the three untyped plasmids. (B) Tra_I-like T4SS of the IncM2 plasmid pKp_SE1_NDM in *K. pneumoniae* strain Kp_SE1_NDM_10_2017 (NZ_CM010662).


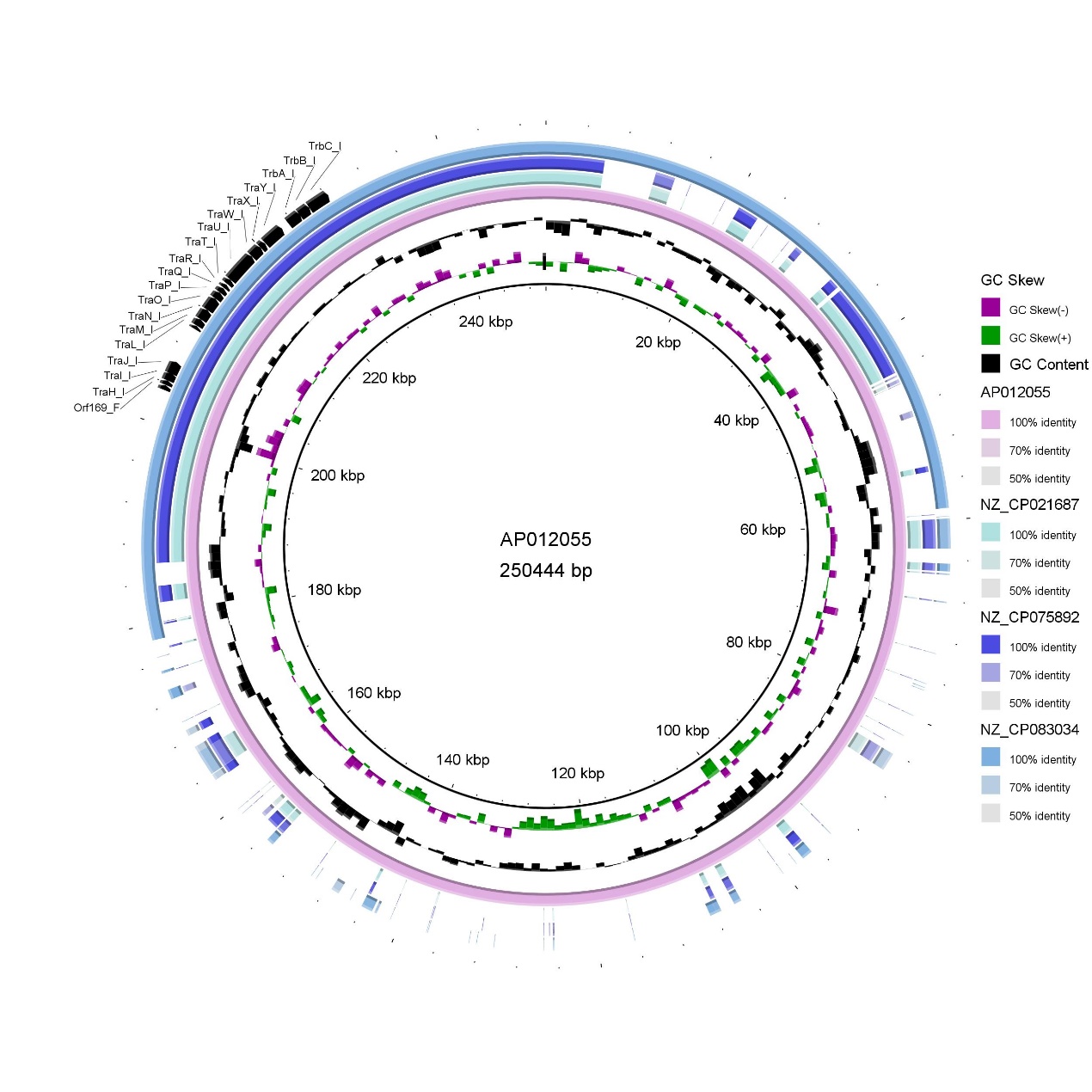


Figure S8. Tra_I-like T4SS gene clusters of plasmid pKPX-1 in *K. pneumoniae* strain KPX (AP012055) and other three plasmids harboring *bla*_NDM_ clustered into clade VII.


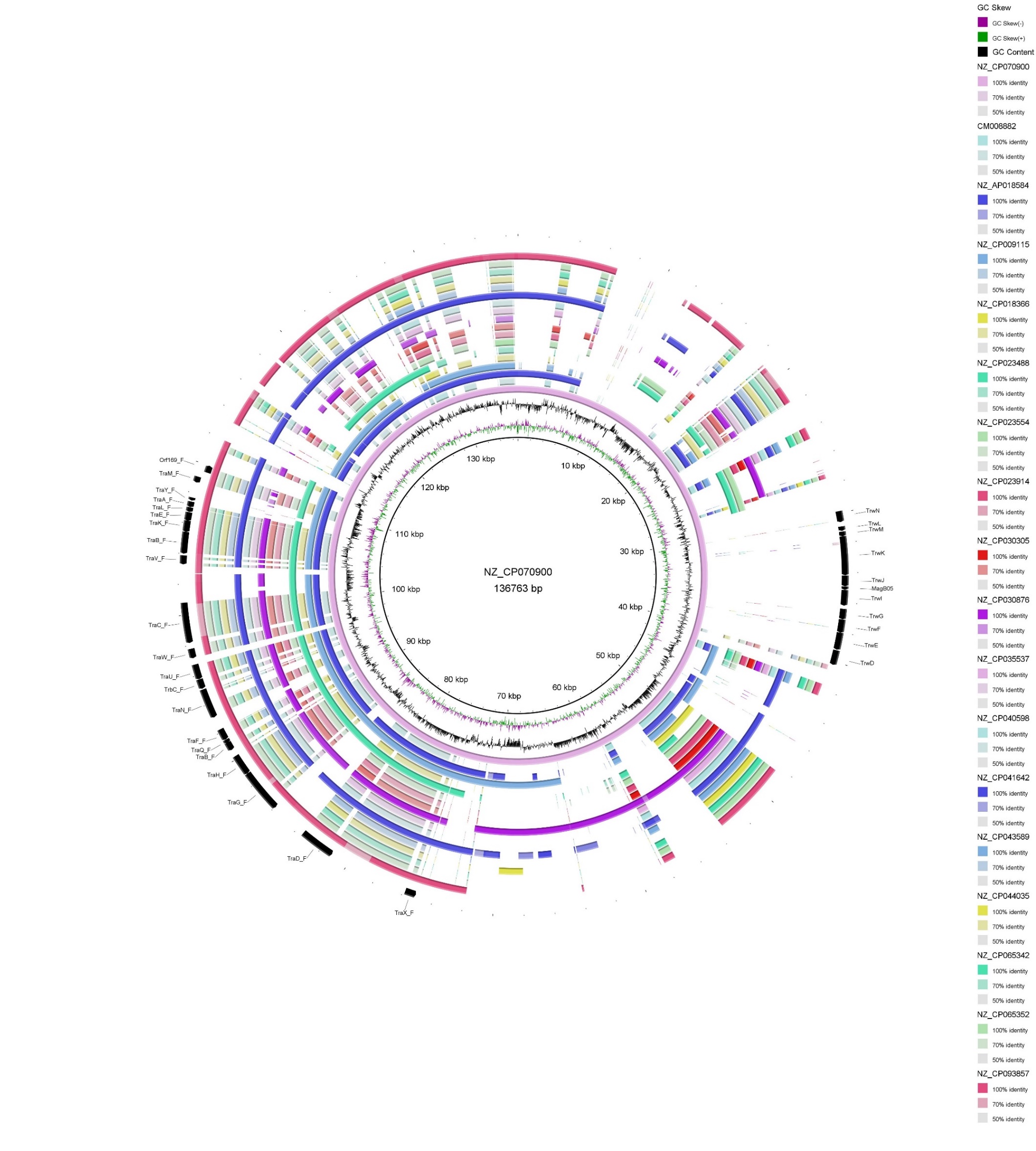


Figure S9. Trw-like T4SS of *K. pneumoniae* strain JNQH116 plasmid pJNQH116-2 (NZ_CP070900) and other 17 IncF plasmids harboring *bla*_NDM_ clustered into clade VIII.


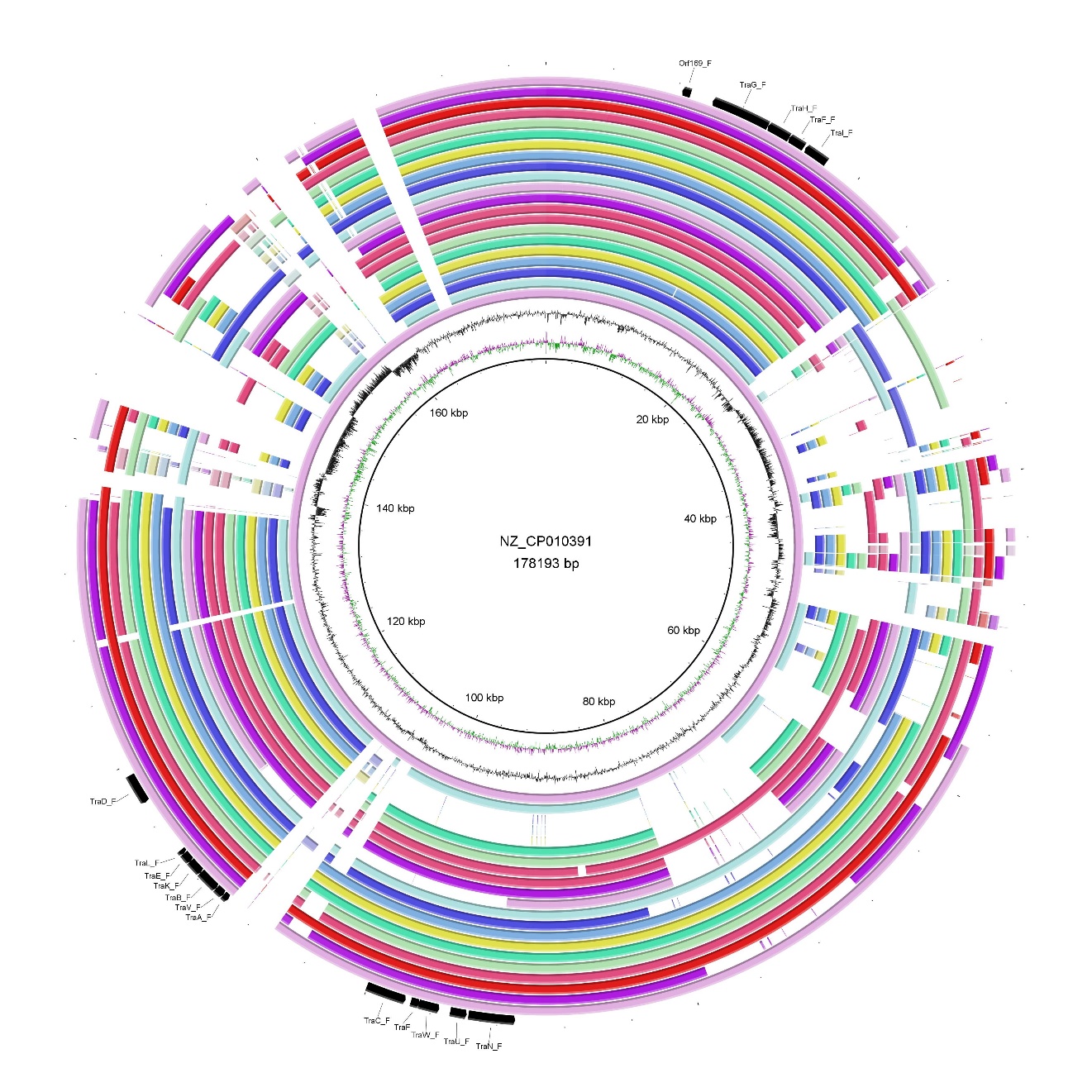
Figure S10. Details of the Tra_F-like T4SS of plasmid p6234-178.193kb in *K. pneumoniae* strain 6234 (NZ_CP010391) and other 20 IncC plasmids harboring *bla*_NDM_ clustered into clade IX.


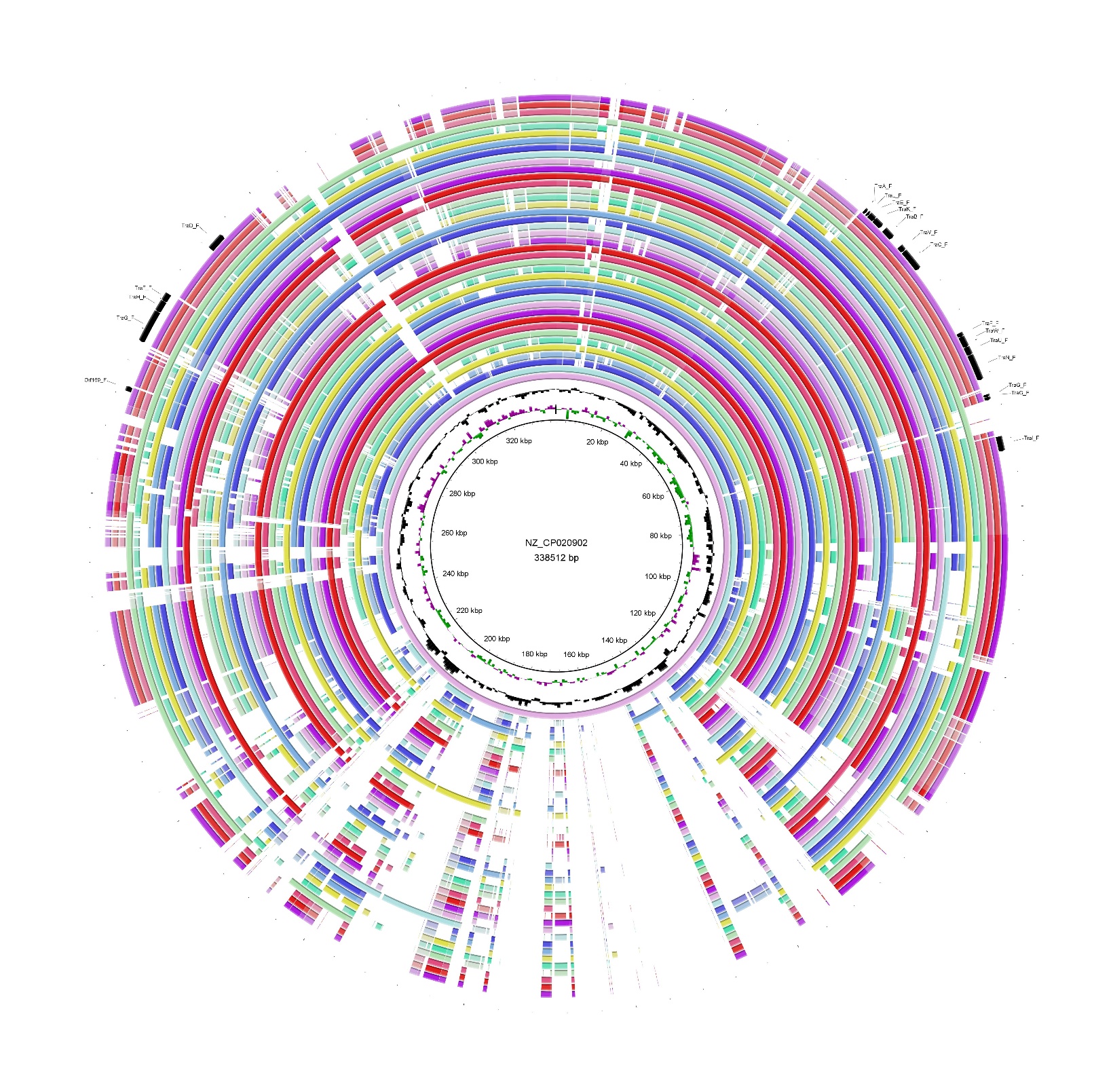


Figure S11. Details of the Tra_F-like T4SS of *K. pneumoniae* strain K66-45 plasmid pK66-45-1 (NZ_CP020902) and other 39 plasmids harboring *bla*_NDM_ clustered into clade X.
